# Erratum for “An ultra‐long‐acting recombinant insulin for the treatment of diabetes mellitus in cats”

**DOI:** 10.1111/jvim.16706

**Published:** 2023-04-07

**First published: 30 June 2021** https://onlinelibrary.wiley.com/doi/10.1111/jvim.16150

**Volume 35, Issue 5**

It has been brought to our attention by a reader that conflict of interest information was missing from the article.

During production, the following conflict of interest declaration provided by the authors at the time of original submission was inadvertently omitted from the final published version of the article: “Thomas Lancaster, Andrea Delpero, Ramya Ragupathy, Sylaja Murikipudi, and Todd Zion are employees and partners in Akston Biosciences. Members of Akston Biosciences were not involved in clinical decision‐making during this study and did not have input on data analysis except for pharmacokinetics data.”

The editors regret this error.

